# Supplementary material for: Patterns of Intron Gain and Loss in Fungi
Source: PLoS Biol. 2004 Nov 30;2(12):e422. doi: 10.1371/journal.pbio.0020422 (PMC532390; doi:10.1371/journal.pbio.0020422)
Supplement: Table S1 — Also available at http://genes.mit.edu/NielsenEtAl/. (4.3 MB ZIP). [file pbio.0020422.st001.zip › NielsenEtAl/html/57.html]

AN0907.1.NCU06431.1.MG06479.1.FG07291.1


```
 CLUSTAL W (1.82) Multiple Sequence Alignments - Introns Inserted


Sequence 1: NCU06431.1	130 aa
Sequence 2: FG07291.1	130 aa
Sequence 3: MG06479.1	130 aa
Sequence 4: AN0907.1	130 aa
Alignment Length: 130 aa
Number Identitical Residues: 103 aa
Alignment Score (without introns) 4279


MG06479.1 	MVRTSVLNDALKSINNAEKAGKRQVMIRPSSKVITKFLSVMQKHG1YIGEFEELDDHRSG
NCU06431.1	MVRTSVLHDALNSINNAEKAGKRQVLIRPSSKVIIKFLQVMQKHG1YIGEFEEVDDHRSG
FG07291.1 	MVRTSVLHDALNAMNNAEKAGKRQVLIRPSSKVIIKFLTVMQRHG1YIGEFEEIDDHRSG
AN0907.1  	MVKTSVLNDALNAINNAEKAGKRQVLIRPSSKVIIKFLSVMQKHG1YIGEFEEVDDHRSG
          	**:****:***:::***********:******** *** ***:** *******:******

MG06479.1 	KIVVQLNGR2LNKTGVISPRYNVRLADLEKWVVKLLPARQFGYVILTTSAGIMDHEEARR
NCU06431.1	KIVVQLNGR~MNKCGVISPRYNVRLAELEKWVVKLLPARQFGYVILTTSAGIMDHEEARR
FG07291.1 	KIVVQLNGR~LNKVGVISPRYNVRLAELEKWVVKLLPARQFGYVILTTSAGIMDHEEARR
AN0907.1  	KIVIQLNGR~LNKCGVINPRYPVQLGDLENWAVQLLPSRQFGFVVLTTSAGIMDHEEARR
          	***:***** :** ***.*** *:*.:**:*.*:***:****:*:***************

MG06479.1 	KHVAGKIIGFFY
NCU06431.1	KHVSGKIIGFFY
FG07291.1 	KHVSGKIIGFFY
AN0907.1  	KHVAGKLLGFFY
          	***:**::****
```
